# A Novel Predictive Score to Identify the Necessity for Epicardial Ventricular Tachycardia Ablation: EPI‐VT‐Score

**DOI:** 10.1111/jce.70061

**Published:** 2025-08-18

**Authors:** Moneeb Khalaph, Denise Guckel, Nadica Trajkovska, Maxim Didenko, Mustapha El Hamriti, Martin Braun, Guram Imnadze, Philip Lucas, Thomas Fink, Vanessa Sciacca, Sebastian Beyer, Yuri Bocchini, Alessandro Guareschi, Arseniy Goncharov, Kawa Mohemed, Volker Rudolph, Christian Sohns, Philipp Sommer

**Affiliations:** ^1^ Department of Electrophysiology, Heart and Diabetes Center NRW Ruhr University Bochum Bad Oeynhausen Germany; ^2^ Clinic for Cardiology/Electrophysiology GZO‐Spital Wetzikon Zürich Suisse; ^3^ Medical Clinic A – Rhythmology University Hospital Ruppin‐Brandenburg Neuruppin Germany; ^4^ Department of Clinical Sciences and Community Health University of Milan Milan Italy; ^5^ Department of General and Interventional Cardiology/Angiology, Heart and Diabetes Center NRW Ruhr University Bochum Bad Oeynhausen Germany

**Keywords:** EPI‐VT‐Score, epicardial VT ablation, indication of VT ablation, VT ablation, VT‐score

## Abstract

**Background:**

Epicardial ventricular tachycardia (VT) ablation is a therapeutic option for drug‐refractory VT, particularly when endocardial ablation fails or is inadequate. However, accurately identifying patients who will benefit most from an epicardial approach remains challenging due to its higher procedure‐related risks.

**Objective:**

This study aimed to develop and validate a predictive scoring model — EPI‐VT‐Score — to identify patients likely to benefit from epicardial VT ablation.

**Methods:**

We retrospectively analyzed data from 138 patients (mean age 64.9 ± 11.3 years, 89.9% male) who underwent VT ablation between 2018 and 2024. Four predictors — underlying cardiomyopathy, left ventricular ejection fraction (LVEF), number of prior VT ablations, and VT‐QRS interval — were identified and incorporated into the EPI‐VT‐Score, which ranges from 4 to 12 points. Score performance was assessed using area under curve (AUC).

**Results:**

Among 138 patients, 51 (37.0%) underwent epicardial ablation. The EPI‐VT‐Score accurately predicted epicardial ablation necessity with an AUC of 0.990 (95% CI, 0.978–1.000). A score ≥ 8 identified epicardial need with 92.2% sensitivity and 100% specificity. Patients scoring < 8 were effectively managed with endocardial‐only ablation.

**Conclusion:**

The EPI‐VT‐Score can be a clinical support to evaluate preprocedural necessity for epicardial access and the complexity of the procedure to improves procedural outcomes as well as minimize unnecessary procedural risks.

## Graphical abstract

The novel EPI‐VT‐Score accurately predicts the need for epicardial VT ablation using simple clinical, echocardiographic and ECG variables, optimizing patient selection and procedural outcomes.

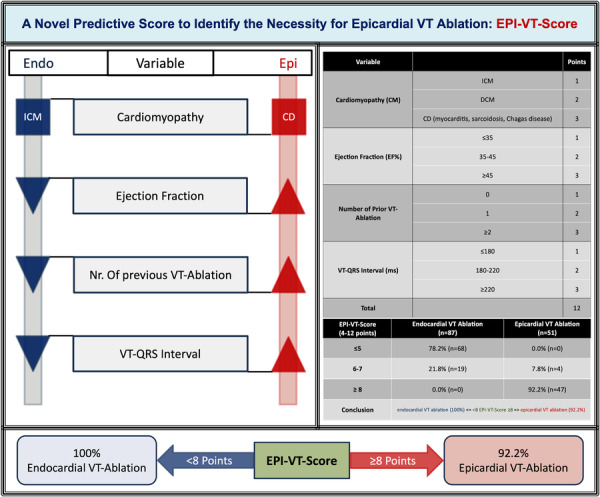

**Abbreviations**
ATP: antitachycardia pacing
AUC: area under curve
CD: cardiac disease
ECG: electrocardiogram
LVEF: left ventricular ejection fraction
MRI: magnetic resonance imaging
VT: ventricular tachycardia

## 1 Introduction

Epicardial ventricular tachycardia (VT) ablation is an advanced therapeutic intervention for managing arrhythmias originating from the outer layer of the myocardium [1, 2]. While most VTs can be successfully treated with an endocardial approach, certain conditions, including arrhythmias arising from epicardial substrates, necessitate an epicardial approach for optimal outcomes [3]. Determining in advance whether a VT ablation will require an endocardial‐only or an additional epicardial approach is crucial, as the latter carries specific implications that electrophysiologists must be aware of. Identifying patients who would benefit from epicardial ablation pre‐procedurally is critical for minimizing unnecessary risks associated with invasive procedures and improving treatment success [4]. The proportion of VT patients necessitating epicardial ablation is highly variable in literature and ranges from 17% to 38% [5, 6].

The absence of a standardized method to predict the need for an epicardial approach before the procedure makes patient selection challenging, highlighting the need for a reliable preprocedural assessment tool. This study aims to create and validate a predictive score —EPI‐VT‐Score— based on preprocedural clinical, echocardiographic and electrocardiographic data.

## 2 Methods

### 2.1 Study Design and Population

This retrospective, cohort study included 138 consecutive patients who underwent VT ablation between 2018 and 2024. Inclusion criteria were patients undergoing ablation for sustained monomorphic VT, with detailed preprocedural data available. Patients with ischemic and nonischemic cardiomyopathy were included. Exclusion criteria included hypertrophic cardiomyopathy and arrhythmogenic right ventricular cardiomyopathy, due to its distinct pathophysiology, predominantly right ventricular substrate, and well‐established indication for epicardial ablation, which could bias the predictive model [7, 8, 9].

### 2.2 Data Collection

Clinical, echocardiographic and electrocardiographic data were collected, including age, body‐mass index, history of underlying cardiomyopathy, left ventricular ejection fraction (LVEF), number of prior VT ablation, procedure parameters and VT‐QRS interval in 12‐lead electrocardiogram (ECG). The procedural need for an epicardial approach was determined based on preprocedural data. This retrospective analysis adhered to the Declaration of Helsinki and compiled with the standards issued by the local ethics committee.

### 2.3 Mapping and Ablation Workflow

Before the procedure, all patients provided written informed consent for catheter ablation, which was performed under general anesthesia. For patients already on oral anticoagulation with a Vitamin K antagonist, the target INR was maintained between 2.0 and 2.5. In patients using direct oral anticoagulants, one dose was withheld immediately before the procedure. Intravenous heparin was administered to maintain an activated clotting time of ≥ 300 s throughout the procedure. Two 7 F sheaths were inserted into the left femoral vein, while an 8.5 F steerable sheath (Carto Vizigo, J&J MedTec, Irvine, USA, or Agilis, Abbott, Chicago, USA) was inserted into the right femoral vein. A decapolar catheter (Inquiry, Abbott, Chicago, USA) was inserted in the distal coronary sinus, and a quadripolar catheter (Supreme, Abbott, Chicago, USA) was positioned at right ventricular apex in all cases.

Left ventricular mapping was performed using the antegrade septal approach only or in combination with a retrograde approach. For the antegrade approach, venous access was obtained via the right femoral vein. Transseptal puncture was performed in the anterior‐inferior portion of the fossa ovalis under fluoroscopic guidance. After transseptal access, high‐density 3D electroanatomical mapping (Carto 3, J&J MedTec, Irvine, USA, or Ensite Precision/X, Abbott, Chicago, USA) of the left ventricle (LV) was performed using a multipolar mapping catheter (PentaRay NAV or OctaRay NAV, J&J MedTec, Irvine, USA, or HD‐Grid, Abbott, Chicago, USA) to assess voltage. Local bipolar voltage < 1.5 mV was defined as low voltage, indicating a potential arrhythmia substrate [10].

For hemodynamically stable monomorphic VT, activation mapping and pacing were performed to identify the critical isthmus location of the VT. In cases of hemodynamically unstable VT, pace mapping was performed at low‐voltage sites identified during sinus rhythm. In patients with frequent ventricular extrasystoles, the earliest ventricular activation on bipolar recordings during clinical PVCs helped identify the origin. Substrate modification was performed based on the voltage and activation maps created. The primary endpoint of catheter ablation was to achieve non‐inducibility of the clinical VT; for this, programmed ventricular stimulation from right ventricular apex was performed using this protocol 500‐, 430‐, 370‐, and 330‐S4 [11]. Finally, if the VT was still inducible especially in case of absence of abnormal endocardial substrate and broad VT‐QRS interval, the decision for epicardial access using a subxiphoid puncture and ablation had been taken.

### 2.4 Score Development: EPI‐VT‐Score

EPI‐VT‐Score was developed based on review of the literature as a predictive tool to assess the likelihood of requiring epicardial access in VT ablation. This score leverages specific clinical, echocardiographic and electrocardiographic parameters that have been identified as predictors of epicardial VT substrate, providing a structured approach to optimize decision‐making, risk stratification and procedural efficiency [1]. The included factors —history of underlying cardiomyopathy, LVEF, number of prior VT ablations and Interval of VT‐QRS on 12‐lead ECG (Table 1)— were selected based on literature evidence and retrospective cohort data, demonstrating a strong correlation with epicardial VT origins [3].

1. **History of Cardiomyopathy:**
  Patients with cardiomyopathies, particularly nonischemic types, are more likely to have epicardial arrhythmic substrates due to fibrotic changes in the epicardium rather than the endocardium [12, 13]. Presence of any form of cardiomyopathy (e.g., myocarditis, sarcoidosis, Chagas disease and less frequently, the dilated cardiomyopathy) is assigned a score between 1 and 3 points, reflecting the increased likelihood of epicardial VT substrates in these patients (Table 1).
2. **Left Ventricular Ejection Fraction (LVEF):**
  Preserved LVEF, particularly over 45%, has been associated with a greater burden of epicardial fibrosis and structural remodeling, increasing the chance of arrhythmic circuits originating from epicardial regions [14, 15]. LVEF < 35% is assigned 1 point and LVEF between 35% and 45% is assigned 2 point while LVEF > 45% assigned 3 points, recognizing the gradient in risk with progressively higher ejection fraction values (Table 1).
3. **Number of prior VT Ablations:**
  Patients who have undergone multiple previous VT ablations are more likely to have persistent or recurrent VT arising from regions inaccessible via prior endocardial ablation, suggesting a possible epicardial source [1]. Each prior VT ablation contributes 1 point to the score, with a maximum of 3 points for this parameter, to reflect the increased likelihood of epicardial substrate with repeated procedural interventions (Table 1).
4. **QRS interval during VT on 12‐Lead ECG:**

**Table 1:** The simple 4 components of preprocedural and its grading for the EPI‐VT‐Score: underlying cardiomyopathy (CM), left ventricular ejection fraction (LVEF), number of prior VT‐ablation and VT‐QRS Interval.

Variable		Points
Cardiomyopathy (CM)	ICM	1
	DCM	2
	CD (myocarditis, sarcoidosis, Chagas disease, etc.)	3
Ejection fraction (EF%)	≤ 35	1
	35–45	2
	≥ 45	3
Number of prior VT‐ablation	0	1
	1	2
	≥ 2	3
VT‐QRS Interval (ms)	≤ 180	1
	180–220	2
	≥ 220	3
Total		12

Abbreviations: CD, cardiac disease; DCM, dilated cardiomyopathy; ICM, ischemic cardiomyopathy.

A prolonged QRS interval, typically > 180 ms, suggests a longer ventricular activation, which is often seen in epicardial VT circuits. This interval reflects a slower propagation of electrical activity through scarred or fibrotic tissue, which is more frequently associated with epicardial arrhythmias [15, 16]. A VT‐QRS interval ≤ 180 ms is assigned 1 point and an interval between 180 and 220 ms is assigned 2 point while an interval ≥ 220 ns is assigned 3 point, acknowledging the graded relationship between VT‐QRS duration and epicardial involvement (Table 1).

### 2.5 Follow up

All patients attended follow‐up visits at 3, 6, and 12 months, including physical exams, ECGs, and device interrogation after the initial procedure. During each visit, they were questioned about any arrhythmia‐related symptoms. Arrhythmia recurrence was defined as any appropriate device‐delivered therapy, including antitachycardia pacing (ATP) or shock.

### 2.6 Statistical Analysis

Data were recorded in Microsoft Office Excel, Version 16.92 (Microsoft, Redmond, WA, USA) (2024). Statistical analysis was performed using SPSS (IBM SPSS Statistics 28). The graphic representation was implemented with Microsoft Office Excel, Version 16.92 (Microsoft, Redmond, WA, USA) (2024). The level of statistical significance was set at *p* ≤ 0.05. Inferential statistics are intended to be exploratory, not confirmatory, and were interpreted accordingly.

The normality of the data distribution was tested using the Kolmogorov–Smirnov test, and the Mann–Whitney U Test was used to compare baseline data. Continuous variables were tested for mean differences using ANOVA. Dichotomous variables were tested with Pearson X2 Test. Fisher's exact test was used to calculate the sensitivities and specificities for the classification report.

## 3 Results

### 3.1 Patients' and Procedural Characteristics

This retrospective study included a total of 138 patients who underwent VT ablation. Among these, 87 (63.0%) underwent endocardial‐only VT ablation, while 51 (37.0%) required both endocardial and epicardial VT ablation.

The mean age was 66.8 ± 9.5 years in the endocardial group and 61.8 ± 13.4 years in the epicardial group. Males accounted for 89.7% of the endocardial group and 90.2% of the epicardial group. The mean LVEF was 29.1 ± 6.5% in the endocardial group and 42.3 ± 10.3% in the epicardial group. The mean total procedural duration was 129.3 ± 25.9 min for endocardial ablation and 191.5 ± 42.5 min for epicardial ablation. The mean total radiofrequency ablation duration was 38.0 ± 12.6 min in the endocardial group and 45.3 ± 17.9 min in the epicardial group. All procedures were performed under fluoroscopic guidance. The average fluoroscopy duration was 6.8 ± 3.9 min for endocardial ablation and 12.3 ± 6.3 min for epicardial ablation. The mean radiation dose was 345.3 ± 300.9 (cGy)*cm² for the endocardial group and 3742.1 ± 3783.7 cGycm² for the epicardial group. The baseline clinical and procedural characteristics are presented in (Table 2).

**Table 2:** The baseline clinical and procedural characteristics.

Variable	Endocardial (*n* = 87)	Epicardial (*n* = 51)	*p* value
Age (yr)	66.8 ± 9.5	61.8 ± 13.4	< 0.05
Male gender (%)	89.7	90.2	0,9
Body‐mass index (kg/m^2^)	27.8 ± 4.2	28.7 ± 4.9	0,3
Ejection fraction (%)	29.1 ± 6.5	42.3 ± 10.3	< 0.05
Arterial hypertension (%)	59.8 (*n* = 52)	60.1 (*n* = 31)	0.9
Antiarrhythmic drugs (%)			
Amiodarone	75.9 (*n* = 66)	80.4 (*n* = 41)	0.5
Sotalol	4.6 (*n* = 4)	9.8 (*n* = 5)	0.3
Implantable cardiac device (%)			
CRT‐D	40.2 (*n* = 35)	31.4 (*n* = 16)	0.3
ICD	59.8 (*n* = 52)	68.6 (*n* = 35)	0.3
Procedure duration (min)	129.3 ± 25.9	191.5 ± 42.5	< 0.05
Fluoroscopy duration (min)	6.8 ± 3.9	12.3 ± 6.3	< 0.05
Fluoroscopy dose (cGy)*cm^2^	345.3 ± 300.9	3742.1 ± 3783.7	< 0.05
Ablation duration (min)	38.0 ± 12.6	45.3 ± 17.9	< 0.05

### 3.2 Score Composition and Predictors

Four significant predictors were identified:

1. History of underlying cardiomyopathy – cardiac Disease (CD) (myocarditis, sarcoidosis, chagas disease, etc.) Associated with increased epicardial involvement (endo: ICM 78.2%, DCM 21.8% and CD 0.0% vs epi: ICM 9.8%, DCM 39.2% and CD 51.0%; *p* < 0.05) (Table 3).
2. Preserved LVEF ‐ Higher prevalence in patients requiring epicardial ablation (endo: EF ≤ 35% 89.7%, EF 35‐45% 8.0% and EF ≥ 45% 2.3% vs epi: EF ≤ 35% 25.5%, EF 35‐45% 23.5% and EF ≥ 45% 51.0%; *p* < 0.05) (Table 3).
3. Number of prior VT ablation – the more, the higher the prevalence for epicardial origin (endo: 0 85.1%, 1 11.5% and ≥ 2 3.4% vs epi: 0 27.5%, 1 39.2% and ≥ 2 33.3%; *p* < 0.05) (Table 3).
4. Broad VT‐QRS interval on ECG ‐ Suggestive of epicardial origin, specifically if matching known epicardial patterns (endo: ≤ 180 ms 62.1%, 180–220 ms 29.9% and ≥ 220 ms 8.0% vs epi: ≤ 180 ms 5.9%, 180–220 ms 31.4% and ≥ 220 ms 62.7%; *p* < 0.05) (Table 3).

**Table 3:** EPI‐VT‐Score components in endo‐ and epicardial VT ablation.

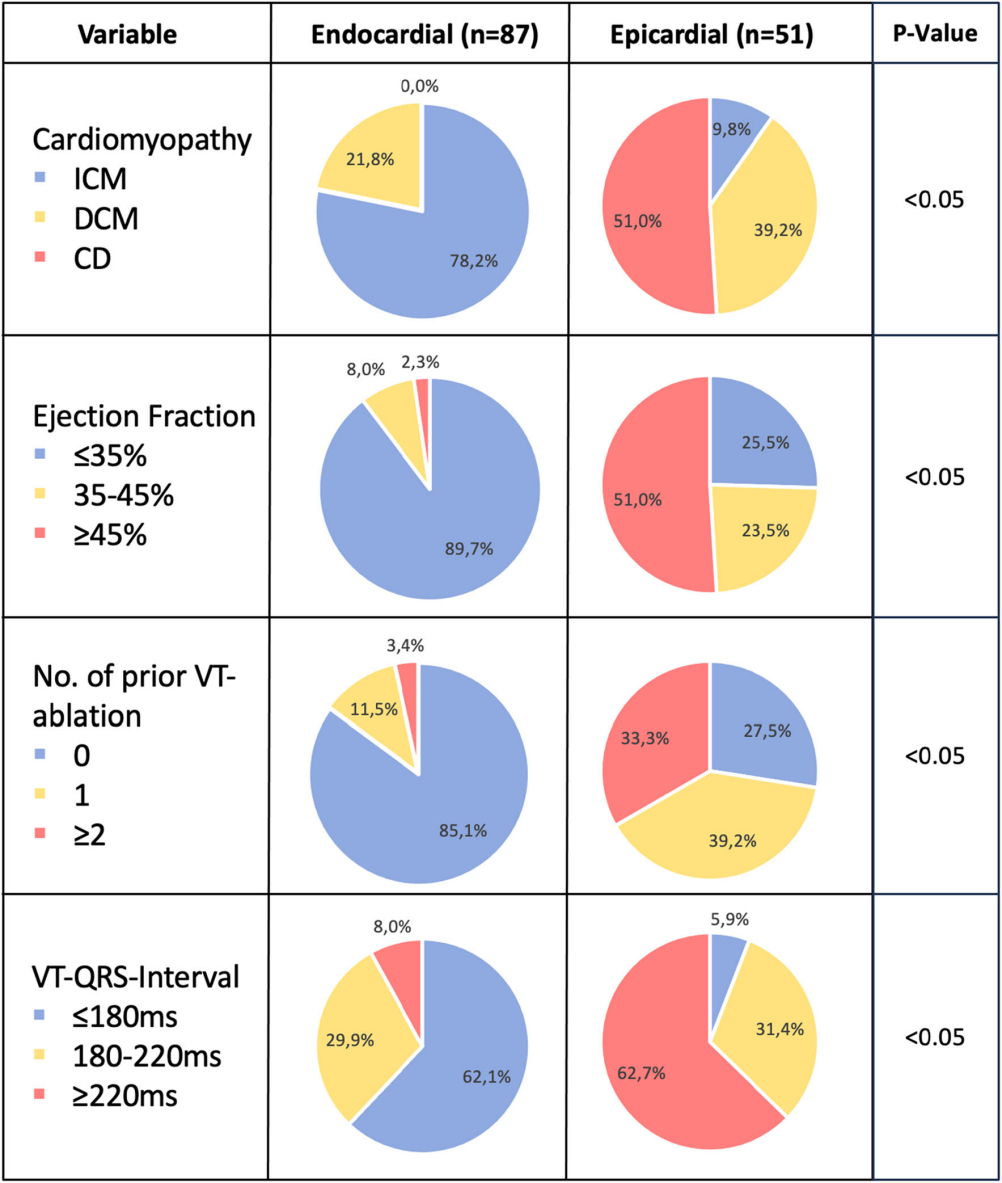

Abbreviations: CD, cardiac disease; DCM, dilated cardiomyopathy; ICM, ischemic cardiomyopathy.

**Figure 1:**
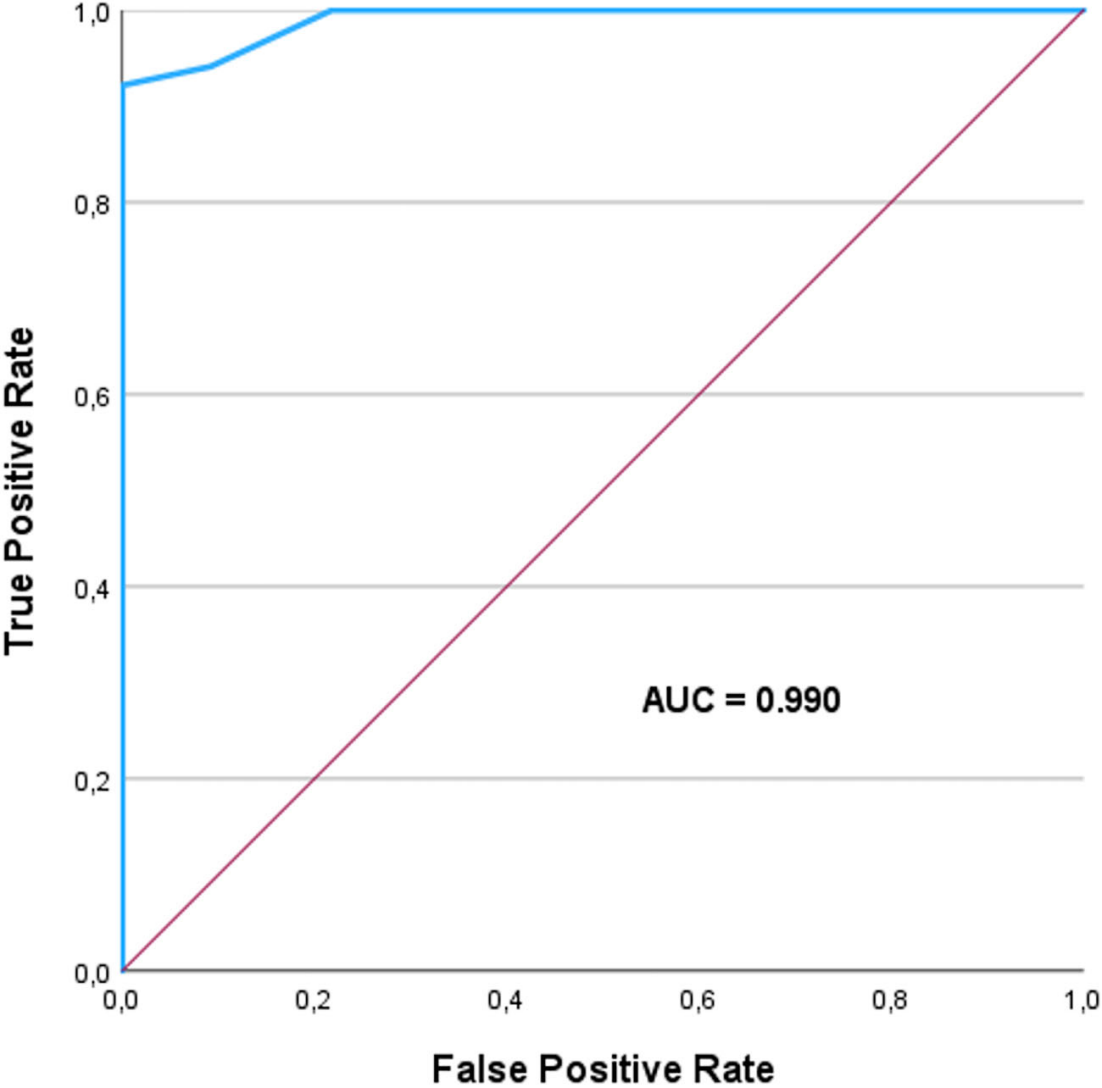
ROC curve: The AUC (area under the curve) is 0.990, indicating excellent performance.

The resulting score had an area under curve (AUC) of 0.990 (95% CI. 0.978–1.000) in the development cohort. A score threshold of ≥ 8 was associated with an 92.2% sensitivity and 100% specificity for predicting the need for epicardial ablation (Figure 1 and Table 4).

**Table 4:** Accuracy of Epi‐VT‐Score in endo‐ and epicardial VT ablation according to the points calculation.

EPI‐VT‐score (4–12 points)	Endocardial VT ablation (*n* = 87)	Epicardial VT ablation (*n* = 51)
** ≤ 5**	78.2% (*n* = 68)	0.0% (*n* = 0)
**6‐7**	21.8% (*n* = 19)	7.8% (*n* = 4)
**≥ 8**	0.0% (*n* = 0)	92.2% (*n* = 47)
**Conclusion**	Endocardial VT ablation (100%) < = < 8 EPI‐VT‐Score ≥ 8 = > epicardial VT ablation (92.2%)	
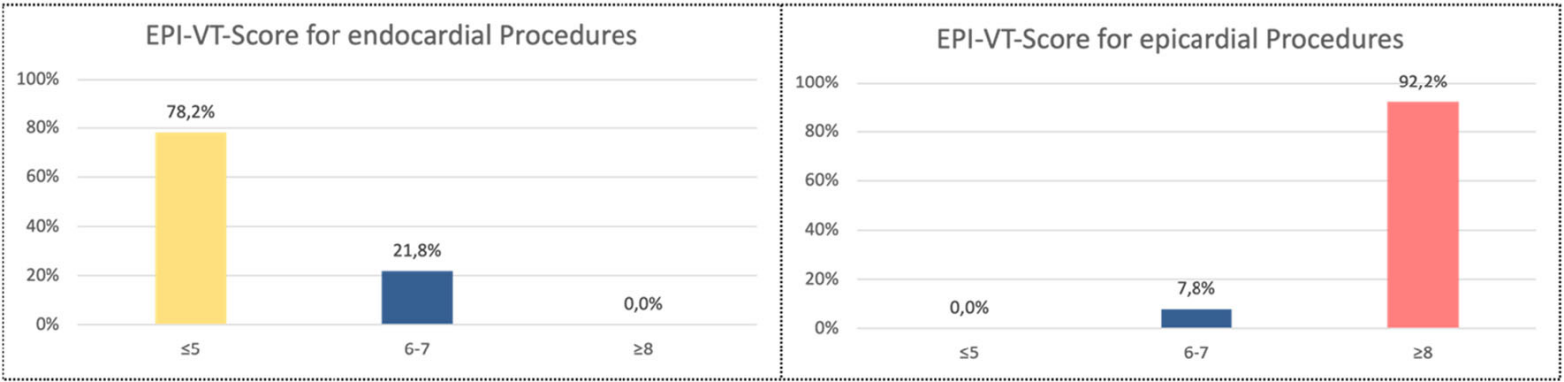		

### 3.3 Interpretation of EPI‐VT‐Score

Each of the four factors was assigned a score based on the strength of its association with epicardial VT. The cumulative EPI‐VT‐Score ranges from 4 to 12, with higher scores indicating a stronger likelihood of requiring an epicardial approach:

− ≤ 5 points: Low likelihood of epicardial substrate; standard endocardial ablation is likely sufficient (EPI‐VT‐Score ≤ 5: endocardial procedures: 78.2% vs epicardial procedures: 0.0%; *p* < 0.05) (Table 4).
− 6–7 points: Moderate likelihood of epicardial substrate; consider epicardial mapping and ablation if endocardial attempts are unsuccessful (EPI‐VT‐Score 6–7: endocardial procedures: 21.8% vs epicardial procedures: 7.8%; *p* < 0.05) (Table 4).
− ≥ 8 points: High likelihood of epicardial substrate; strongly consider an initial epicardial approach or combined endo‐epicardial strategy (EPI‐VT‐Score ≥ 8: endocardial procedures: 0.0% vs epicardial procedures: 92.2%; *p* < 0.05) (Table 4).

The score demonstrated a stepwise increase in epicardial ablation rates with higher point totals, supporting its discriminatory capacity across clinical scenarios.

### 3.4 Follow‐up

Patients were followed for 12 months post ablation to assess recurrence of VT and procedural outcomes. The recurrence rate in the epicardial ablation group was 25%, while the endocardial group showed a recurrence of 31%, underscoring the importance of accurate patient selection for epicardial intervention.

## 4 Discussion

The development and validation of the EPI‐VT‐Score represent a significant advancement in the preprocedural assessment for VT ablation. This study highlights the importance of integration specific clinical, echocardiographic and electrocardiographic parameters, such as underlying cardiomyopathy, LVEF, history of number of prior VT ablations, and VT‐QRS interval on 12‐lead ECG [17], to better predict the necessity of an epicardial approach for optimal treatment outcomes. Given the known increased procedural risks associated with epicardial ablation [1, 12, 15], including hemopericardium, phrenic nerve injury, coronary arteries damage and pericardial complications [4, 18], Accurate preprocedural assessment is crucial. EPI‐VT‐Score provides a strategic tool to identify patients most likely to benefit from an epicardial approach, thereby reducing unnecessary procedures and potential complications. This scoring system aims to provide a straightforward preprocedural tool to enhance procedural planning, reduce the need for repeat ablations, and improve clinical outcomes for VT patients with epicardial substrates. In cases where epicardial ablation is indicated, early recognition allows for better procedural planning, ensuring appropriate resources such as cardiothoracic surgical backup are available to enhance patient safety.

### 4.1 Clinical Implications of EPI‐VT‐Score

The identified predictors in EPI‐VT‐Score, including underlying cardiomyopathy and LVEF, are well‐supported by existing literature as markers of an increased likelihood of epicardial VT origin [13, 14]. Specifically, inclusion the reason of cardiomyopathy, particularly in patients with nonischemic etiologies, reflects previous findings that these patients often exhibit arrhythmias driven by epicardial substrates due to the unique distribution of fibrotic tissue in the epicardium [19]. Furthermore, LVEF, particularly when preserved, was associated with a higher probability of epicardial VT, aligning with studies that show such patients often have areas of patchy fibrosis conducive to epicardial reentry circuits [20]. The identification of multiple prior VT ablations as a predictor also addresses an important clinical observation: recurrent VT post‐endocardial ablation often originates from previously untreated epicardial regions, thus warranting an epicardial approach [1, 17].

### 4.2 Imaging and its Exclusion From EPI‐VT‐Score

Advanced imaging modalities such as cardiac magnetic resonance imaging (MRI) and computed tomography are highly valuable in VT ablation planning, particularly for identifying epicardial or intramural substrates in nonischemic cardiomyopathy [21, 22, 23]. However, imaging was not included in the EPI‐VT‐Score to maintain simplicity and ensure broad applicability.

Nevertheless, retrospective MRI data support the score's predictive value. Among the 51 patients in the epicardial ablation group, cardiac MRI was available in 25 patients (49%). Of these, all exhibited epicardial or transmural late gadolinium enhancement (LGE), suggesting the presence of epicardial substrate. Specifically, epicardial LGE was present in 100%, intramural in 40%, and endocardial in only 8% of cases. These findings align with the score's ability to identify patients likely to require epicardial ablation.

While EPI‐VT‐Score supports rapid and straightforward clinical decision‐making, imaging remains an important complementary tool in VT ablation planning. Future prospective studies incorporating imaging findings may further validate and refine the score.

### 4.3 Comparison With Existing Predictive Models

While other scores exist for risk stratification in VT management [24, 25], few have specifically targeted the decision‐making process for epicardial access [1, 3, 26]. Most conventional scores are focused on general VT recurrence and patient mortality risk without consideration of substrate location [27]. EPI‐VT‐Score fills this critical gap by providing clinicians with a tailored tool to assess the necessity for epicardial intervention, thus bridging a divide in current arrhythmia management protocols.

### 4.4 Utility in Clinical Practice and Future Research

The high predictive accuracy of EPI‐VT‐Score, demonstrated by an AUC of 0.990, suggests that this tool can be effectively used in routine clinical practice. By identifying patients with a score of ≥ 8 as high likelihood candidates for epicardial ablation, EPI‐VT‐Score offers a clear and actionable threshold for procedural planning. Moreover, this score can potentially decrease the number of repeat procedures, as accurate initial assessment can improve success rates by directing candidates toward an appropriate epicardial approach when necessary.

Moreover, the score can be easily integrated into the clinical workflow, as it is simple to calculate, relies on readily available patient data, and does not require dedicated software or invasive testing.

Future research should explore the score's applicability in a multicenter setting to ensure generalizability across diverse patient populations and procedural environments. Additionally, prospective studies with long‐term follow‐up are warranted to assess how the score influences patient outcomes, recurrence rates, and overall procedural success in a real‐world context.

Furthermore, since no standardized scoring systems currently exist for epicardial VT ablation, a direct comparison with current decision‐making, could further underscore the advantages of the EPI‐VT‐Score, highlighting its potential to refine procedural planning and optimize patient selection.

### 4.5 Limitations

This study has several limitations. Its retrospective design may limit generalizability, and the relatively small sample size reduces the robustness of the findings. The focus on specific clinical, echocardiographic, and electrocardiographic parameters excluded other potentially influential factors, such as detailed myocardial scar imaging and comorbidities. Additionally, the determination of VT‐QRS interval cutoff points and scoring was based on the available sample, and further validation in larger cohorts is necessary to refine these thresholds. While the EPI‐VT‐Score showed high accuracy, external validation in larger, prospective, multicenter cohorts is needed to confirm its applicability.

## 5 Conclusion

EPI‐VT‐Score is a promising tool for preprocedural prediction of epicardial VT ablation indication, integrating key clinical, echocardiographic and electrocardiographic parameters to aid in procedural planning and optimize patient outcomes. By targeting patients who are most likely to benefit from an epicardial approach, EPI‐VT‐Score holds the potential to improve procedural efficacy, minimize unnecessary risks, and contribute meaningfully to personalized arrhythmia management strategies. Prospective validation in larger cohorts, along with a direct comparison to current clinical decision‐making strategies, will be crucial to confirm its clinical utility and adaptability across different patient populations.

## Author Contributions

C.S. received research support and lecture fees from Medtronic, Abbott, Boston Scientific, and J&J MedTech; is a consultant for Medtronic, Boston Scientific, and J&J MedTech; has received grant support from the Else Kröner‐Fresenius‐Stiftung and Deutsche Herzstiftung. P.S. is Member of Advisory Board for Abbott, Boston Scientific, J&J MedTech and Medtronic. All other authors state: None.

## Data Availability

Data will be made available from the authors on reasonable request.
